# Identification of Hub Genes and Therapeutic Agents for IgA Nephropathy Through Bioinformatics Analysis and Experimental Validation

**DOI:** 10.3389/fmed.2022.881322

**Published:** 2022-06-28

**Authors:** Ming Xia, Di Liu, Haiyang Liu, Liang Peng, Danyi Yang, Chengyuan Tang, Guochun Chen, Yu Liu, Hong Liu

**Affiliations:** Hunan Key Laboratory of Kidney Disease and Blood Purification, Department of Nephrology, The Second Xiangya Hospital, Central South University, Changsha, China

**Keywords:** IgA nephropathy, hub gene, bioinformatics, tetrandrine, mesangial cell

## Abstract

**Background:**

IgA nephropathy (IgAN) is the most common primary glomerular disease and the leading cause of the end-stage renal disease in the world. The pathogenesis of IgAN has not been well elucidated, and yet treatment is limited. High-throughput microarray has been applied for elucidating molecular biomarkers and potential mechanisms involved in IgAN. This study aimed to identify the potential key genes and therapeutics associated with IgAN using integrative bioinformatics and transcriptome-based computational drug repurposing approach.

**Methods:**

Three datasets of mRNA expression profile were obtained from the gene expression omnibus database and differentially expressed genes (DEGs) between IgAN glomeruli and normal tissue were identified by integrated analysis. Gene ontology and pathway enrichment analyses of the DEGs were performed by R software, and protein-protein interaction networks were constructed using the STRING online search tool. External dataset and immunohistochemical assessment of kidney biopsy specimens were used for hub gene validation. Potential compounds for IgAN therapy were obtained by Connectivity Map (CMap) analysis and preliminarily verified *in vitro*. Stimulated human mesangial cells were collected for cell proliferation and cell cycle analysis using cell counting kit 8 and flow cytometry, respectively.

**Results:**

134 DEGs genes were differentially expressed across kidney transcriptomic data from IgAN patients and healthy living donors. Enrichment analysis showed that the glomerular compartments underwent a wide range of interesting pathological changes during kidney injury, focused on anion transmembrane transporter activity and protein digestion and absorption mostly. Hub genes (*ITGB2, FCER1G, CSF1R*) were identified and verified to be significantly upregulated in IgAN patients, and associated with severity of renal lesions. Computational drug repurposing with the CMap identified tetrandrine as a candidate treatment to reverse IgAN hub gene expression. Tetrandrine administration significantly reversed mesangial cell proliferation and cell cycle transition.

**Conclusion:**

The identification of DEGs and related therapeutic strategies of IgAN through this integrated bioinformatics analysis provides a valuable resource of therapeutic targets and agents of IgAN. Especially, our findings suggest that tetrandrine might be beneficial for IgAN, which deserves future research.

## Introduction

IgA nephropathy (IgAN) is the most common primary glomerular disease, with an incidence of about 25 patients per 1 million people in the population and the highest incidence in the Asia Pacific region (1, 2). It is a frequent cause of end-stage renal disease (ESRD), progressive glomerular and interstitial sclerosis leads to end-stage kidney failure in 20–40% of patients within 20 years after diagnosis, which brings heavy disease and economic burden to the individual and the whole society (3). A kidney biopsy is required for IgAN diagnosis which is characterized by mesangial immune deposits of IgA1/galactose-deficient IgA1 (Gd-IgA1) with C3 and occasionally IgG or IgM (4, 5). To date, multiethnic genome-wide association studies involving over 20,000 individuals have identified risk loci predisposing to IgAN, highlighting the importance of innate and adaptive immunity in this disorder (6). Published gene chips and high-throughput sequencing also give clues to the pathogenesis of IgAN, while there is still a lack of clear understanding of the pathogenesis of IgAN and short of effective therapeutics for disease progression. In-depth exploration of the public datasets, combined with laboratory validation, may reveal associated risk genes.

Connectivity Map (CMap) is one of the first publicly available tools for drug repurposing, utilizing transcriptomics to form unbiased connections between diseases, genes, and drugs (7). These connections can identify drugs that reverse large numbers of genes disturbed in disease (8). In this study based on bioinformatics, three IgAN datasets were integrated from the GEO database and performed differentially expressed genes (DEGs), gene ontology (GO) and kyoto encyclopedia of genes and genomes (KEGG) pathway analyses. The protein-protein interaction (PPI) network was performed, and hub genes with high connectivity in network that are expected to play an important role in understanding the biological mechanisms of IgAN were identified, and were further validated with external and laboratory data. We also interrogated the CMap with disease-related DEGs and identified tetrandrine as a reversal agent, and examined the impact of tetrandrine on human mesangial cells. Overall, our output suggest potential key genes and novel therapeutic agents for IgAN.

## Materials and Methods

### Acquisition of Microarray Data and DEGs

The microarray data were downloaded from the gene expression omnibus (GEO) database (http://www.ncbi.nlm.nih.gov/geo) using the keywords “IgA nephropathy,” “Homo sapiens” and “Expression profiling by array” were included in the next round of screening. The gene expression microarray datasets GSE104948 (9), GSE93798 (10) GSE37463 (11) were selected and downloaded for DEG analysis. Probes were matched to the gene symbols using the annotation files provided by the manufacturer. DEGs were identified by sva/limma R package (12) (version 4.1.0) after consolidation and batch normalization, with threshold |log_2_FC| >1 and adjusted *p* < 0.05. Heatmap (https://CRAN.R-project.org/package=pheatmap) and volcano plot were subsequently performed using R software to visualize the DEGs.

### Bioinformatic DEG Analysis

GO enrichment and KEGG pathway analyses were performed to explore the potential biological processes and utilities, cellular components, and molecular functions of DEGs using colorspace (13) and stringi (https://stringi.gagolewski.com/) R packages. Significantly relevant signal pathways were identified via clusterProfiler package (14) in R software with p <0.05 and q < 0.05. The STRING database (https://string-db.org) (15) was used to explore potential protein-protein interactions (PPI) interactions between DEGs, and a network interaction matrix was built. An interaction with a combined score > 0.7 was set as the cut-off value. Densely connected clusters in the PPI network were visualized by R.

### External Microarray Dataset and Laboratory Validation

To validate the expression of hub genes in other glomerulonephritis types and IgAN, we use GSE116626 (16) as an independent validate cohort, comprising 52 IgAN patients, 22 non-IgAN glomerulonephrites (non-IgAN GN), and 7 kidney living donors (LD). The relative mRNA expression levels of hub genes in each patient were extracted from the raw data, which were then analyzed by Graphpad Prism 6. Statistical differences were analyzed by Student's *t*-test, and *p* < 0.05 was considered statistically significant. Analyses were conducted with GraphPad Prism 6.0 statistical software.

### Immunohistochemistry Staining

Formaldehyde-fixed and paraffin-embedded tissue sections (4 mm thick) were used for immunohistochemistry as previously described (17). After dehydration, slides in citrate solution were subjected to a microwave antigen retrieval process. Primary antibodies were employed against the integrin beta 2 (ITGB2) (Santa Cruz Biotechnology, sc-8420), fc fragment of IgE receptor Ig (FCER1G) (Santa Cruz Biotechnology, sc-390222), and colony-stimulating factor 1 receptor (CSF1R) (Santa Cruz Biotechnology, sc-46662), periodic acid-Schiff (PAS) was used instead of primary antibodies as negative controls for all staining. Finally, horseradish peroxidase (HRP)-conjugated polymer (Abcam, USA) was used for the visualized detection under light microscopy (Nikon Tokyo, Japan). OD values were analyzed by ImageJ software (National Institute of Health, USA). The use of renal specimens of IgAN patients was approved by the Ethics Committee of the Second Xiangya Hospital of Central South University (2019SNK1222000) according to the Declaration of Helsinki.

### CMap Analysis

The CMap (https://portals.broadinstitute.org/cmap) is an open resource that links disease, genes, and drugs by similar or opposite gene expression profiles (18), and was used to explore potential drugs targeting IgAN. Upregulation and the downregulation DEGs were converted to probe sets in the chip type to query the CMap. A list of CMap instances predicted to reverse IgAN DEGs was obtained, with connectivity score and *p*-value.

### Experimental Cell Culture and Treatment

The human mesangial cells (HMCs) collected from (CBR130735, Cellbio, China) were cultivated in Dulbecco's Modified Eagle's Medium (DMEM)/F-12 medium (Gibco, USA) supplemented with 10% fetal bovine serum (FBS) under 5% CO_2_ at 37 °C. Monomeric human IgA1 (Abcam, ab91020) was heated and aggregated at 65°C for 150 min on a dry plate heater to obtain aggregated IgA1 (aIgA1) as previously described (19). After cooling at room temperature, the proteins were centrifuged at 11,000g for 5 min to remove insoluble precipitant. The supernatants were then used as heat-aggregated IgA. Mesangial cells were treated with or without IgA aggregates at a final concentration of 25 μg/ml for 24 h. The dose of IgA aggregates was selected based on our previous study in mesangial cells (17). Tetrandrine (10 mM in 1 ml dimethyl sulfoxide) was purchased from ApexBio Technology (Houston, USA). Cells were incubated with aIgA1 alone or with a combination of tetrandrine (0–10 μM) concentrations for 24 h.

### Cell Proliferation Assay

Cell proliferation was accessed by cell counting kit-8 (CCK8) assay (Dojindo, Japan) following the manufacturer's recommended procedure. 10 μl of CCK8 was added to treated cells in 96-wells and incubated at 37 °C. The absorbance was detected at 450 nm using spectrophotometer.

### Cell Cycle Analysis

Cell cycle was measured by cell cycle and apoptosis kit (Wellbiology, China), and the treated cells were fixed overnight with 70% cold ethanol at 4°C, then stained with a mixture of propidium iodide and RNase A at 37°C for 30 min. The distribution of the cell cycle phase was measured by FlowJo software.

## Results

### Data Information and DEGs Identification

**Figure 1:**
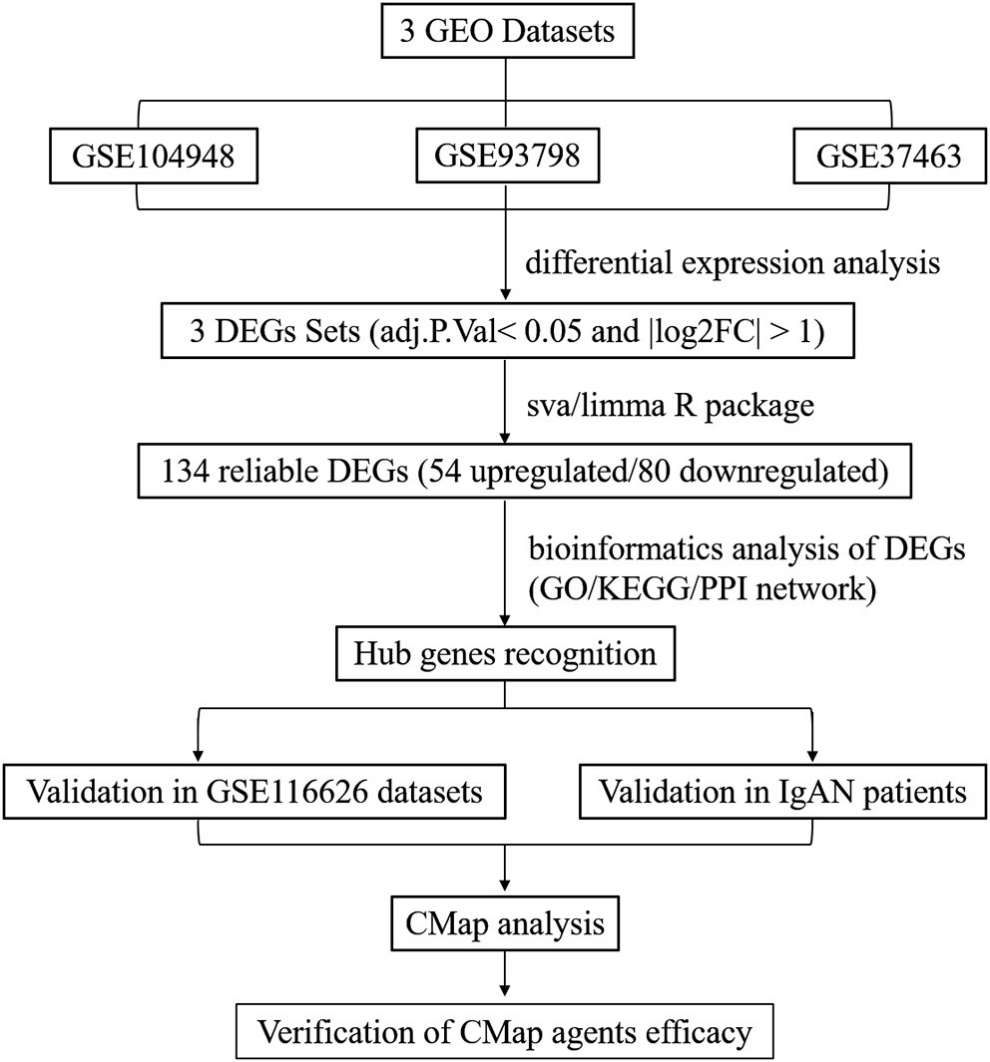
Flowchart presenting the process of identification of potential hub genes and therapeutic agents in IgAN.

**Figure 2:**
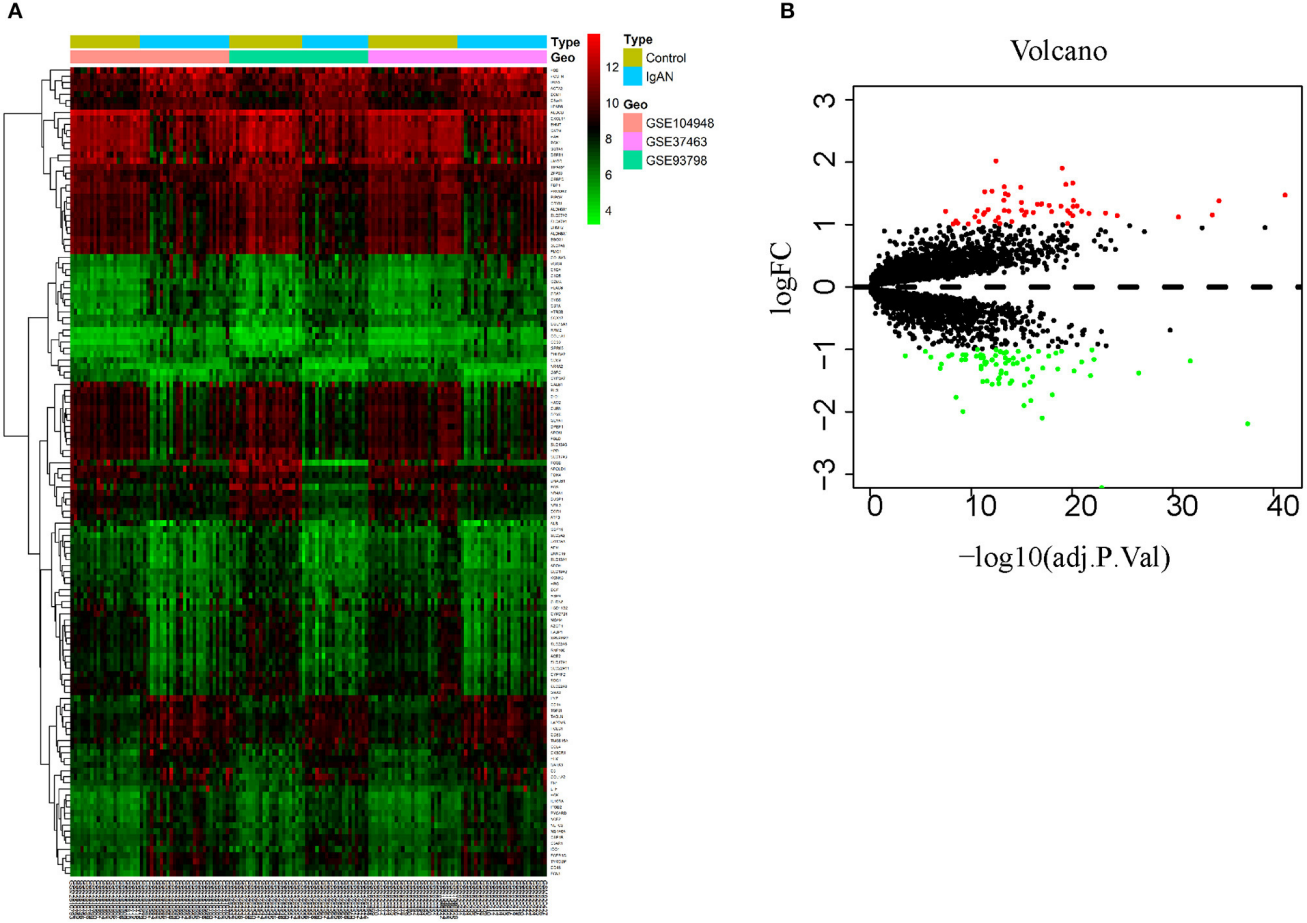
Identification of DEGs between IgAN and normal tissues. **(A)** Heat map of 134 DEGs identified by integrated analysis of the GEO datasets. Red areas represent highly expressed genes and green areas represent lowly expressed genes in glomeruli from IgAN subjects compared with healthy living donors. **(B)** Volcano plot analysis identifies DEGs. Red dots represent 54 upregulated genes and green dots represent 80 downregulated genes in glomeruli from IgAN subjects compared with healthy living donors.

This study was conducted according to the flow chart shown in Figure 1. The microarray data archives for glomeruli of IgAN were searched and downloaded from the Gene Expression Omnibus (GEO) (https://www.ncbi.nlm.nih.gov/geo/) using the “IgA nephropathy,” with “Homo sapiens” and “Expression profiling by array” filters in the next round of screening. Three relevant microarray expression profiles (GSE104948, GSE93798, GSE37463) were selected between IgAN and normal glomeruli compartment. A total of 144 human glomerular compartment tissue samples were screened, of which 74 were IgAN patients and 70 were healthy donors. Details of the GEO datasets in this study were shown in Table 1. A total of 134 DEGs were identified after consolidation and batch normalization (Figure 2A; Supplementary Table S1), including 54 upregulated and 80 downregulated genes. The volcano plot (Figure 2B) showed the distribution of DEGs between IgAN and normal controls.

**Table 1:** Details of the included GEO datasets in the study.

**Datasets**	**References**	**Platform**	**Included sample (Normal/IgAN)**	**Application**
GSE104948	Grayson et al. (9)	GPL22945, GPL24120	21/27	Identification of DEGs
GSE93798	Liu et al. (10)	GPL22945	22/20	Identification of DEGs
GSE37463	Berthier et al. (11)	GPL11670, GPL14663	27/27	Identification of DEGs
GSE116626	Cox SN et al. (16)	GPL14951	7/52	Validation

### DEGs Functional Enrichment Analysis

**Figure 3:**
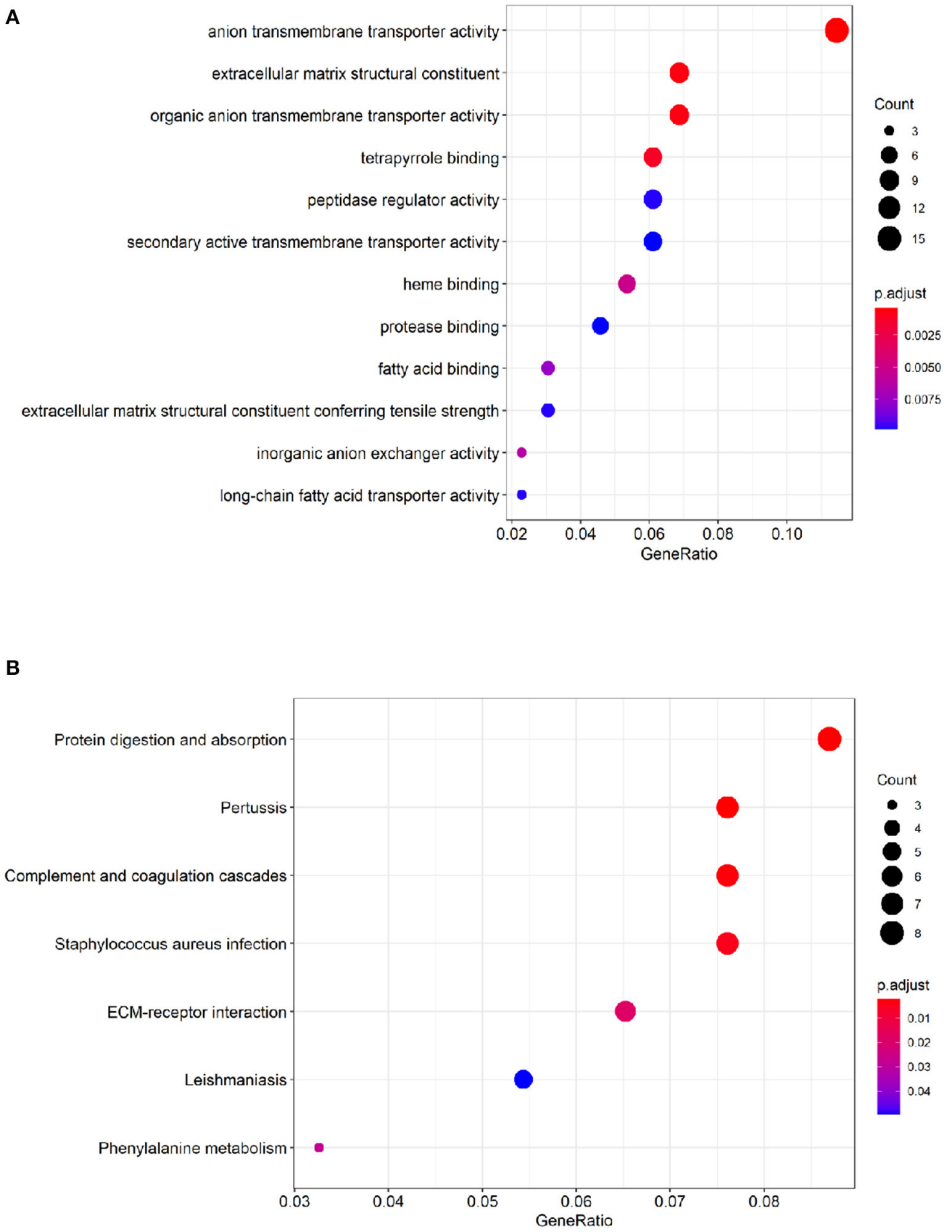
Functional enrichment analysis of the DEGs. **(A)** Top 12 GO terms of the DEGs. **(B)** 7 enriched KEGG pathways of the DEGs. The color depth of the nodes refers to the adj *P*-value. The size of the nodes refers to the number of genes.

To identify comprehensive information on biological processes, cellular components, and molecular functions gene function, GO and KEGG pathway analyses of DEGs were performed. The top twelve GO terms with the highest degree of enrichment were shown in Figure 3A. GO terms mainly concentrated on anion transmembrane transporter activity, extracellular matrix structural constituent, and organic anion transmembrane transporter activity. Figure 3B presented seven enriched KEGG pathways of the DEGs, protein digestion and absorption, pertussis, complement, and coagulation cascades were identified as the top three significantly relevant signal pathways.

### PPI Network Construction and Hub Genes Recognition

**Figure 4:**
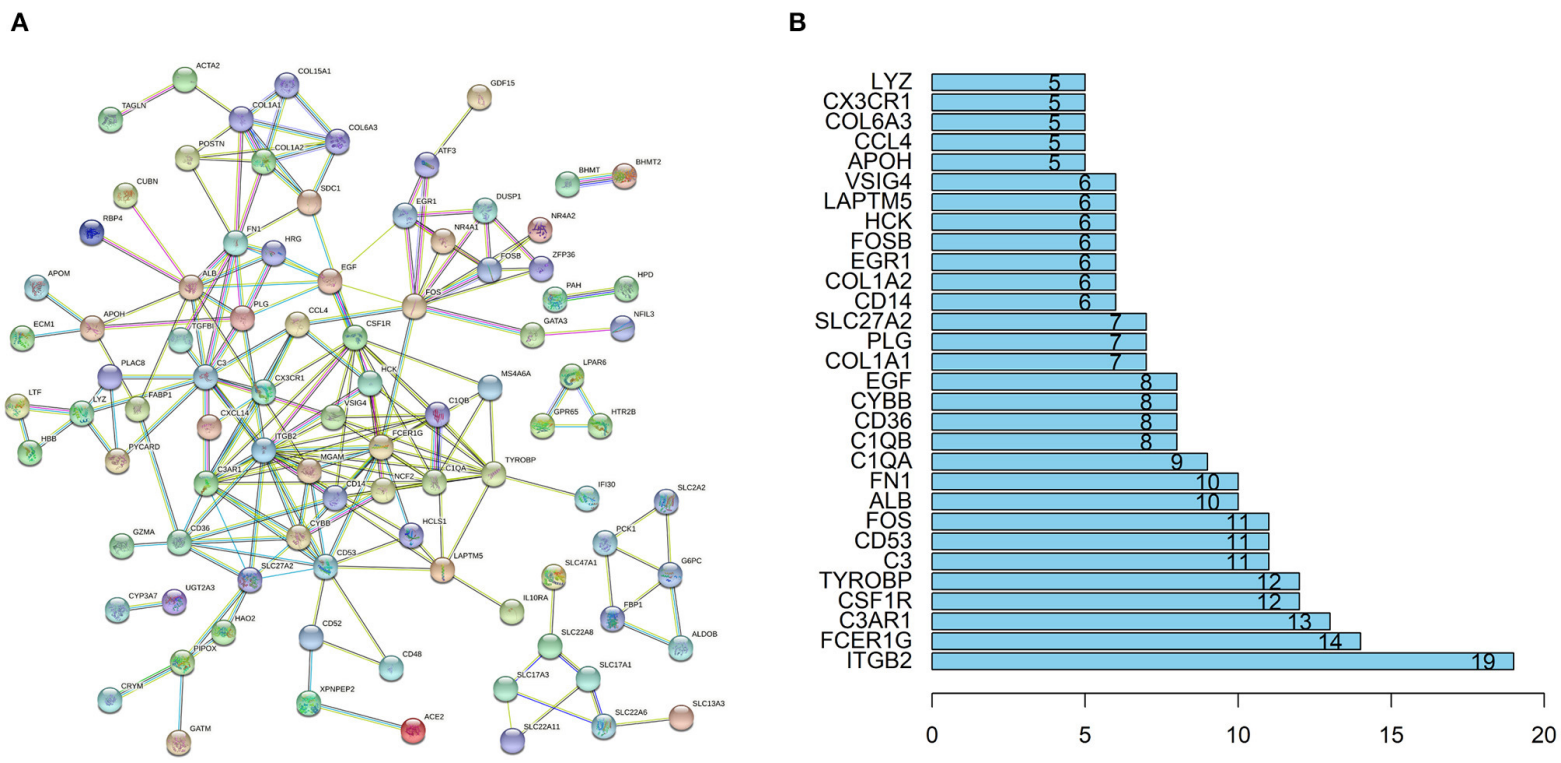
PPI network and the hub genes of DEGs. **(A)** The PPI network of DEGs was constructed using String with high confidence. **(B)** The top 30 key genes were obtained from the PPI network. The numbers on the bar represent the number of related nodes for each gene.

The STRING database (https://string-db.org) was used to explore potential interactions between DEGs. High confidence score>0.7 serves as the cut-off point for the construction of protein-protein interaction (PPI) network, visualization of the PPI network was shown in Figure 4A. A total of 91 nodes and 194 interactions were calculated by R software, the top 30 candidate hub genes which may play a central role in this network were identified in Figure 4B. Five genes with the highest score were obtained as *ITGB2, FCER1G*, complement C3a receptor 1 (*C3AR1*), *CSF1R*, transmembrane immune signaling adaptor TYROBP (*TYROBP)*.

### External Validation of Hub Genes

**Figure 5:**
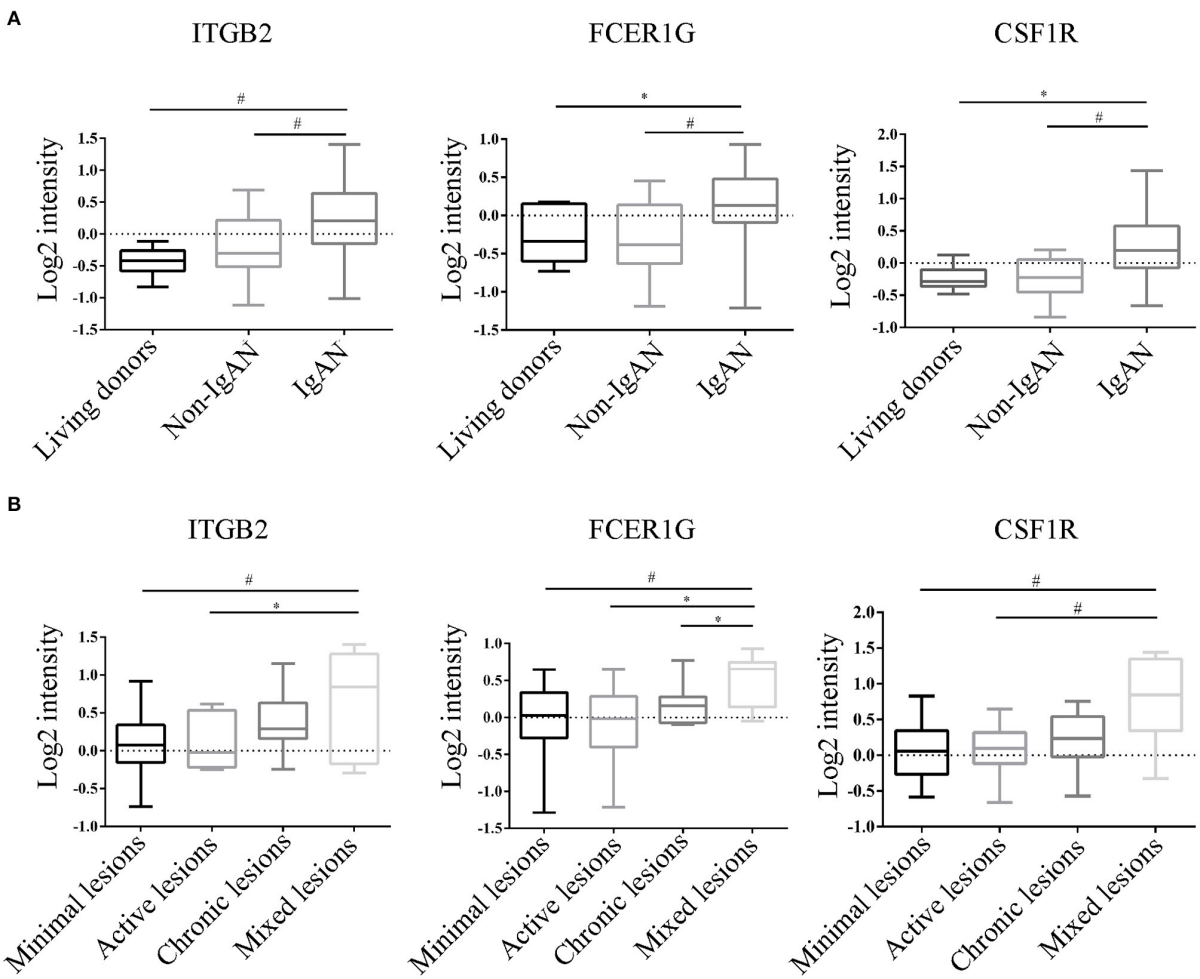
Transcriptional levels were analyzed in kidney biopsy specimens of living donors, non-IgAN, and IgAN patients in the GSE116626 dataset. **(A)** Levels of *ITGB2, FCER1G, CSF1R* in living donors, non-IgAN, and IgAN patients. **(B)** Levels of *ITGB2, FCER1G, CSF1R* with minimal, active, chronic, and mixed renal lesions in IgAN. *p < 0.05, ^#^*p* < 0.01.

**Figure 6:**
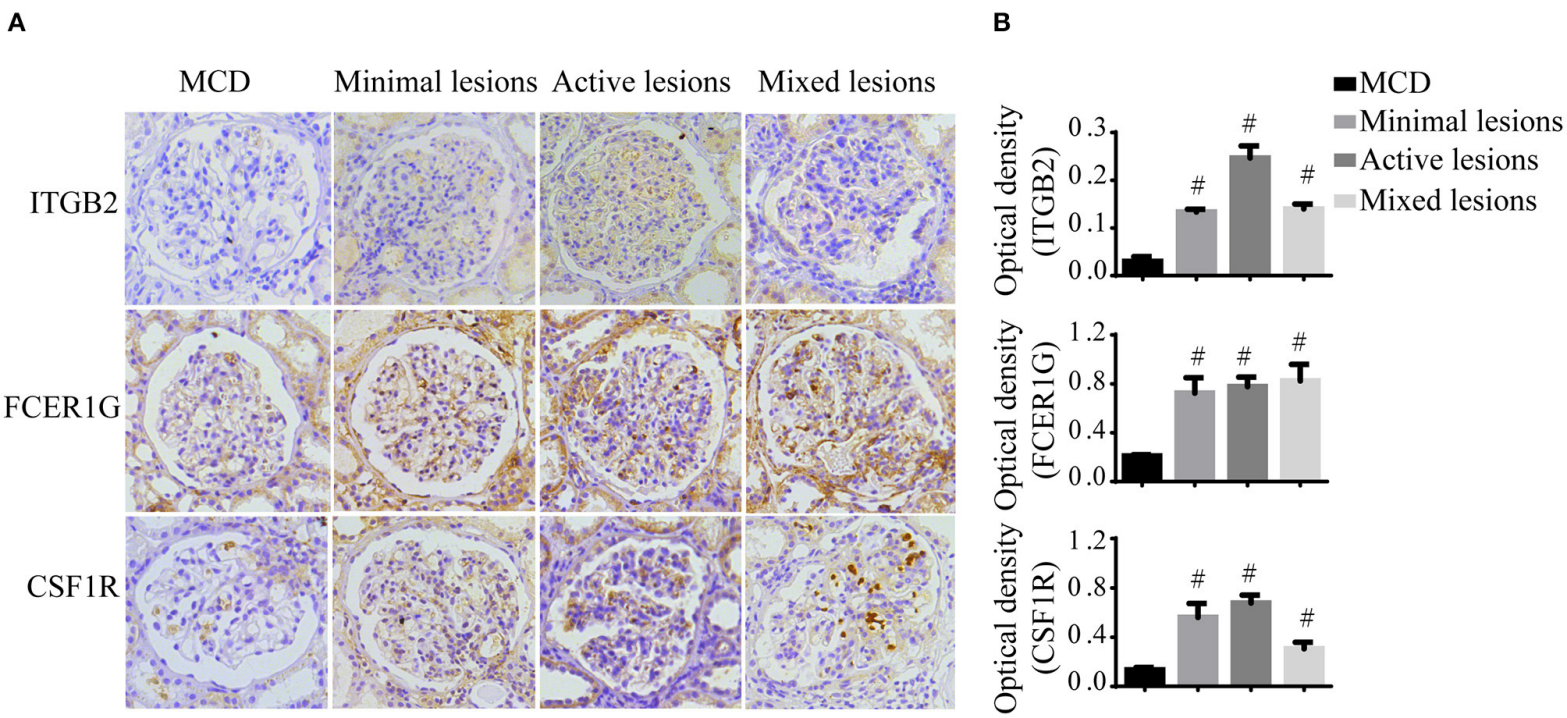
Immunohistochemistry of ITGB2, FCER1G, and CSF1R in IgAN and MCD kidneys. **(A)** Representative images of ITGB2, FCER1G, and CSF1R glomerular immunohistostaining from MCD and IgAN patients with the minimal lesion, active lesion, mixed lesions. **(B)** The average optical density of ITGB2, FCER1G, and CSF1R was analyzed by ImageJ software. Data were presented as mean ± SD. Magnification 40×. ^#^*p* < 0.01 vs. MCD. MCD, minimal change disease.

To verify the reliability of the data and the link between key genes and IgAN, an external dataset GSE116626 (16), which was uploaded by Cox et al. in 2018 and updated in 2020, was used to validate the top 5 hub genes. *ITGB2, FCER1G, C3AR1, CSF1R, TYROBP* expression profiles were screened in archival FFPE biopsy samples of 52 IgAN patients, 22 non-IgAN glomerulonephrites (non-IgAN GN), and 7 kidney living donors (LD). The transcriptional levels of *ITGB2, FCER1G*, and *CSF1R* were significant higher in IgAN compared to either healthy donors or non-IgAN (Figure 5A), while *C3AR1* and *TYROBP* transcriptional levels were not significant (data not shown). We further investigated the *ITGB2, FCER1G*, and *CSF1R* transcriptional and protein levels in IgAN with different composite renal lesions, patients were stratified into 4 groups based on the E, C,and T (but not M or S) scores: minimal (E0, C0, T0); active (E1 and/or C1, C2, T0); chronic (T1 or T2, E0, C0);mixed group composed of active and chronic lesions (E1 and/or C1, C2, T1 or T2) as previously described (16). Compared with minimal lesions or active lesions, these three genes mRNA expression were significantly increased in the mixed lesions group. Also, *FCER1G* mRNA level was significantly upregulated in the mixed lesion group compared to chronic lesions (Figure 5B). Moreover, biopsy specimens of minimal change disease (MCD) and IgAN were collected in our hospital. Through immunohistochemistry, ITGB2, FCER1G, and CSF1R were significantly elevated in IgAN patients with different renal pathology compared with MCD (Figure 6).

### CMap Analysis

**Figure 7:**
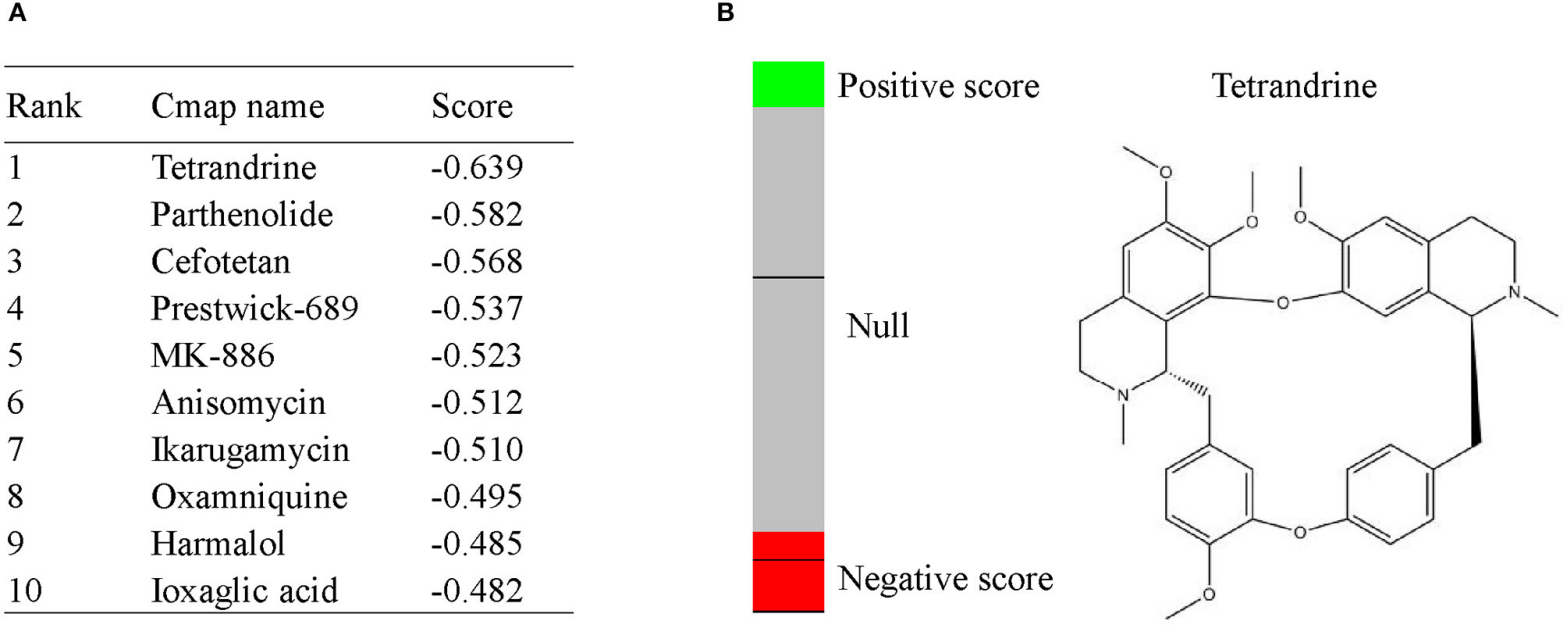
CMap analysis indicated potential treatment options for IgAN. **(A)** Top10 CMap compounds predicted to reverse altered DEGs expression. **(B)** The bar graph represents the connectivity score data for tetrandrine. The black horizontal lines represent each instance performed with the respective compound. Instances in the red area indicate negative correlation scores, and instances in the green area indicate positive ones. No correlation could be detected for instances in the gray area. The chemical structure of tetrandrine was shown.

To screen potential therapeutic small molecular compounds for IgAN, we performed CMap (http://portals.broadinstitute.org/cmap/) analysis and obtained a list of drug candidates predicted to reverse altered expression of DEGs (Figure 7A). Tetrandrine, an antirheumatic and analgesic agent, was the top-ranked candidate with the highest negative connectivity score (Figure 7B) and was selected for subsequent validation experiments.

### Effect of Tetrandrine on Mesangial Cells Proliferation

**Figure 8:**
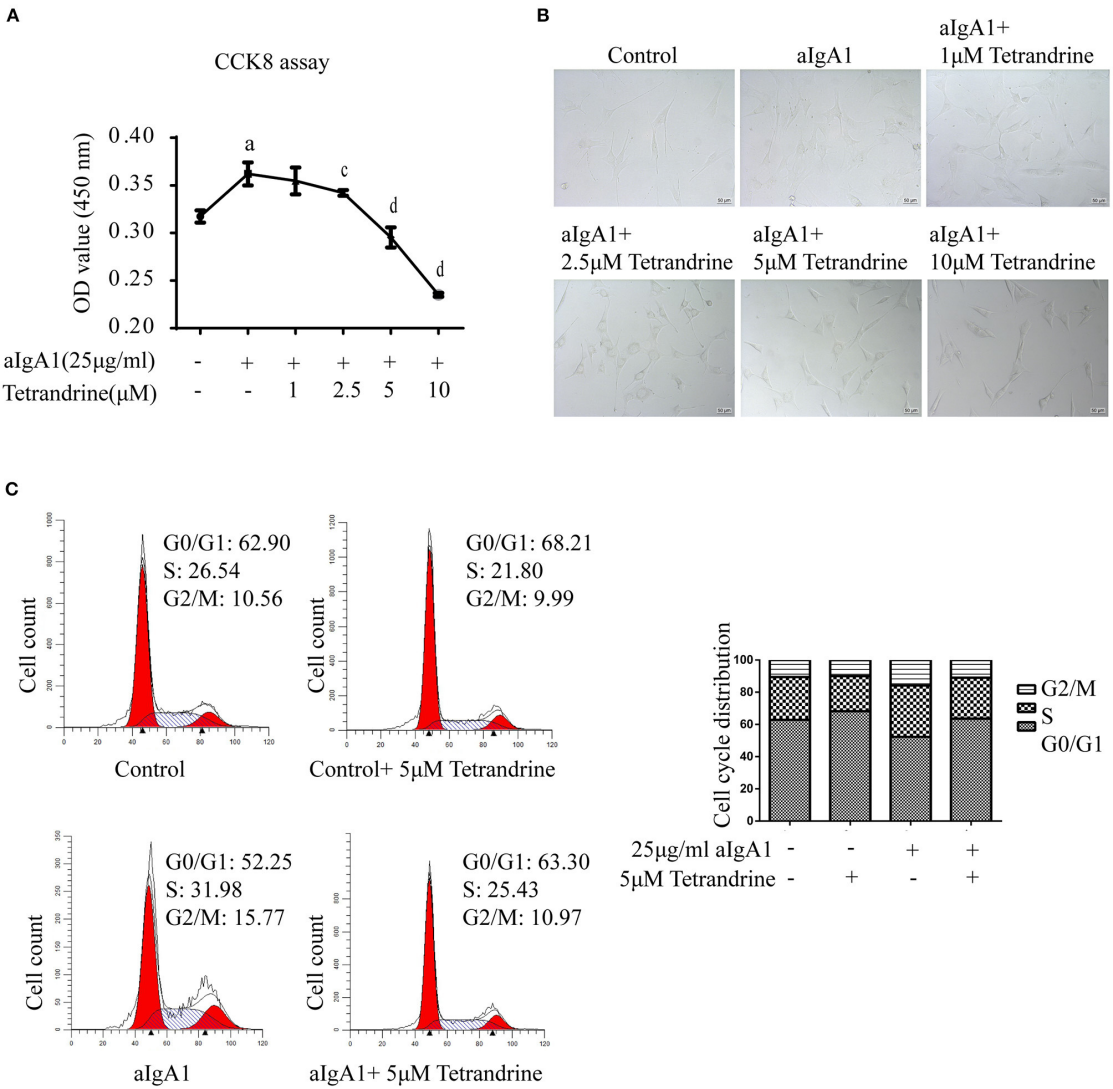
Effect of tetrandrine on the proliferation of HMCs. **(A)** Cell proliferation of aIgA1-induced HMCs under 1–10 μM tetrandrine treatment was detected by CCK8 assay. Data were presented as mean ± SD (*n* = 3 in each group). **(B)** Representative light microscopy images were presented. **(C)** The cell cycle of HMCs treated with 25 μg/ml aIgA1 and 5 μM tetrandrine was detected by flow cytometry. ^a^*p* < 0.05 vs. control; ^c^*p* < 0.05, ^d^*p* < 0.01 vs. aIgA1 group.

Considering that mesangial cell proliferation is a prominent pathological feature of IgAN, to further investigate the renal protective effect of tetrandrine, we focused its role on the proliferation of human mesangial cells (HMCs). 25 μg/ml aIgA1-stimulated HMCs was used to mimic the IgAN HMC model *in vitro*, as previously described (17). The CCK8 assay showed that tetrandrine at concentrations of 2.5–10 μM for 24 h had strong inhibitory effects on aIgA1-induced mesangial proliferation, and did not alter the viability of HMCs (Figure 8A). The representative light microscopy was shown in Figure 8B. To further confirm the effect of tetrandrine on the proliferation of HMCs, cell cycle analysis was carried out by flow cytometry. After 24 h incubation with aIgA1, the proportion of cells in S and G2/M phases increased, while 5 μM tetrandrine retained more cells in G0/G1 phase (Figure 8C).

## Discussion

IgAN was recognized as a separate category of glomerulonephritis when Berger & Hinglais applied the immunofluorescence staining technique to renal biopsy in 1968. Much progress has occurred in understanding immunologic and biochemical defects underlying IgAN, but there is a paucity of effective treatments for IgAN. Here, we presented a transcriptome-based drug repurposing approach identifying potential treatments for IgAN.

First, we described IgAN DEGs using combined analyses of three glomeruli microarray data, since glomerular injury is better associated with a progressive decline of kidney function than tubule cell activation and injury. Interestingly, extracellular matrix structural constituent and complement and coagulation cascades were observed in functional enrichment analyses, represented genes were fibronectin (FN1) which was revealed to aid in IgA deposition (20), and C3, a verified complement colocalized with IgA deposition (21, 22). Our previous report also showed that coagulation parameters prothrombin time (PT) and the activated partial thromboplastin time (APTT) were associated with the prognosis of IgAN (23).

Second, we screened three hub genes-*ITGB2, FCER1G*, and *CSF1R* and validated them in an additional published dataset and our patients. These genes were reported to be enhanced in immune cells (macrophage) and demonstrated a prominent inflammatory signature, reflecting increased infiltrating local inflammation in IgAN. *ITGB2* encodes the integrin beta chain, which combines with multiple different alpha chains to form different integrin heterodimers. It participates in cell surface-mediated signaling as well as immune response, and defects in this gene cause leukocyte adhesion deficiency (24). ITGB2 has also been reported to be negatively correlated with eGFR in patients with CKD (25) and involved in cell adhesion and extracellular matrix remodeling in renal cancer (26). *FCER1G* is located on chromosome 1q23.3 and encodes the cytoplasmic Fc receptor gamma chain (FcRg) of immunoglobulin. It is a signal-transducing subunit containing an immunoreceptor tyrosine-based activation motif (ITAM) that transduces activation signals from various immunoreceptors that play a critical role in allergic reactions, immune cell activation, and chronic inflammatory programs (27, 28). It has been illustrated that FCER1G participated in various kidney diseases, such as diabetic kidney disease and clear cell renal cell carcinoma (29, 30). Recently FCER1G was identified as a marker of inflammatory dendritic cells and abundantly expressed in the periglomerular region of the lupus nephritis kidney (31). *CSF1R* encodes a tyrosine-protein kinase that acts as a cell-surface receptor for CSF1 and IL-34 and regulates the production and differentiation of most circulating/tissue-resident macrophages. Activation of CSF1R in the kidney could induce monocyte recruitment from the blood and proliferation and survival of tissue-resident macrophages, as well as skew macrophages toward an M2 phenotype (32), *CSF1R* knockout mice significantly reduced macrophages (33). Another bioinformatics analysis has also predicted that *ITGB2, FCER1G* and *CSF1R* were involved in the development and progression of IgAN (34), but our study is the first to verify this in biopsy specimens and explore related therapeutics. In addition, our results indicated that *ITGB2, FCER1G* and *CSF1R* were highly expressed in IgAN glomeruli and in mesangial cells of frozen IgAN sections, which may contribute to recruit monocytes/macrophages and further amplifying inflammatory responses. It has been reported that galactose deficient-IgA1 isolated from IgAN patient serum could initiate podocyte macrophage transdifferentiation (35), we speculate that mesangial cells may also have similar transformation characteristics and need further exploration.

Third, we applied our disease characteristics to CMap, which has the advantage that most of the recorded compounds have already been approved by regulators, so the benefits demonstrated in experimental models could be quickly translated into human disease (7, 36). After screening more than 1,000 compounds through CMap pattern-matching algorithms linked IgAN hub genes to drugs that could likely reverse them based on empirical evidence, tetrandrine was identified as the top candidate. With molecular formula C_38_H_42_N_2_O_6_ and weight 622.3, tetrandrine has been used in the treatment of T cell lymphoma, rheumatism, and silicosis in China for several decades (37, 38). Tetrandrine has been reported to possess extensive pharmacological properties including anticancer (39), anti-inflammation (40, 41), immunomodulatory effects (42), and regulates several important biological processes in the pathogenesis of kidney diseases. Tetrandrine could alleviate podocyte injury induced by TRPC6 overexpression (43), decrease 24 h urine protein and glomerular cell proliferation in the membranous glomerulopathy (44), and restrain diabetic process and renal damage in diabetic nephropathy (45). Early studies have shown that tetrandrine could inhibit the activation of mesangial cells by down-regulating the ERK/ NF-κB signaling pathway (46), suggesting a protective effect on the pathological proliferated mesangial cells mediated renal damage. Here, consistent with other reports of cell cycle regulation (47–50), tetrandrine was observed to significantly inhibit cell cycle transition at G1/S boundary as well as aIgA1-induced mesangial cells proliferation. Further comprehensive investigation of the therapeutic effect and mechanism of tetrandrine on IgA nephropathy is warranted.

In conclusion, we identified differential expression genes between kidneys of IgAN and healthy controls, and validated the hub genes *ITGB2, FCER1G*, and *CSF1R* closely associated with IgAN renal damage. CMap analysis provided tetrandrine as a potential therapeutic compound to reverse the disease signature, possibly due in part to its renal protective effects by inhibiting cell cycle transition and proliferation of HMC. Together, our outputs could empower the novel potential targets and treatment to IgAN therapy.

## Data Availability Statement

The datasets presented in this study can be found in online repositories. The names of the repository/repositories and accession number(s) can be found in the article/Supplementary Material.

## Ethics Statement

The studies involving human participants were reviewed and approved by Ethics Committee of the Second Xiangya Hospital of Central South University. The patients/participants provided their written informed consent to participate in this study.

## Author Contributions

MX contributed to study design, experiments, and draft the manuscript. DL, HYL, and LP contributed to data collection and statistical analysis/interpretation. DY contributed to pathological experimental techniques. CT, GC, and YL contributed to review and editing. HL gave the final approval for the article to be published. All authors have read and approved the final manuscript.

## Funding

This work was supported by the National Natural Science Foundation of China (82070737), Hunan Provincial Natural Science Foundation of China (2021JJ30940), Hunan Provincial Innovation Foundation for Postgraduate (CX20210368), and Fundamental Research Funds for the Central Universities of Central South University (2021zzts0366).

## Conflict of Interest

The authors declare that the research was conducted in the absence of any commercial or financial relationships that could be construed as a potential conflict of interest.

## Publisher's Note

All claims expressed in this article are solely those of the authors and do not necessarily represent those of their affiliated organizations, or those of the publisher, the editors and the reviewers. Any product that may be evaluated in this article, or claim that may be made by its manufacturer, is not guaranteed or endorsed by the publisher.
