# Supplementary material for: Identification of Hub Genes and Therapeutic Agents for IgA Nephropathy Through Bioinformatics Analysis and Experimental Validation
Source: Front Med (Lausanne). 2022 Jun 28;9:881322. doi: 10.3389/fmed.2022.881322 (PMC9273898; doi:10.3389/fmed.2022.881322)
Supplement: Supplementary file 1 [file Table_1.docx]

**Table S1. 134 differentially expressed genes between IgAN and normal controls.**

| **Gene symbol** | **LogFC** | **Adj.P.Val** | **Description** |
| --- | --- | --- | --- |
| HBB | 2.0188 | 3.49E-13 | Hemoglobin Subunit Beta |
| COL1A2 | 1.9054 | 9.49E-20 | Collagen Type I Alpha 2 Chain |
| CX3CR1 | 1.6664 | 8.13E-21 | C-X3-C Motif Chemokine Receptor 1 |
| TYROBP | 1.6456 | 4.04E-20 | Tyro Protein Tyrosine Kinase Binding Protein |
| LYZ | 1.6083 | 5.21E-14 | Lysozyme |
| HTR2B | 1.5976 | 1.22E-15 | 5-Hydroxytryptamine Receptor 2B |
| C1QB | 1.5364 | 8.47E-13 | Complement C1Q B Chain |
| COL6A3 | 1.5250 | 4.51E-12 | Collagen Type Vi Alpha 3 Chain |
| FCN1 | 1.4930 | 4.13E-14 | Ficolin 1 |
| C1QA | 1.4783 | 2.19E-14 | Complement C1Q A Chain |
| C8orf4 | 1.4730 | 7.51E-42 | Chromosome 8 Open Reading Frame 4 |
| ECM1 | 1.3913 | 5.64E-14 | Extracellular Matrix Protein 1 |
| IL10RA | 1.3875 | 7.36E-21 | Interleukin 10 Receptor Subunit Alpha |
| HLX | 1.3795 | 2.71E-35 | H2.0 Like Homeobox |
| CD52 | 1.3558 | 1.04E-15 | Cd52 Molecule |
| HCK | 1.3330 | 1.00E-17 | Hck Proto-Oncogene, Src Family Tyrosine Kinase |
| CD53 | 1.3276 | 2.62E-17 | Cd53 Molecule |
| TGFBI | 1.3267 | 1.13E-17 | Transforming Growth Factor Beta Induced |
| CD48 | 1.3079 | 1.85E-18 | Cd48 Molecule |
| IFI30 | 1.2995 | 3.17E-21 | Ifi30 Lysosomal Thiol Reductase |
| FN1 | 1.2969 | 1.26E-19 | Fibronectin 1 |
| FCER1G | 1.2918 | 7.66E-21 | Fc Fragment Of Ige Receptor Ig |
| RRM2 | 1.2405 | 1.98E-12 | Ribonucleotide Reductase Regulatory Subunit M2 |
| POSTN | 1.2317 | 9.71E-16 | Periostin |
| CD36 | 1.2270 | 5.26E-14 | Cd36 Molecule |
| CD14 | 1.2236 | 2.79E-20 | Cd14 Molecule |
| VSIG4 | 1.2200 | 2.54E-11 | V-Set And Immunoglobulin Domain Containing 4 |
| PLAC8 | 1.2157 | 1.86E-14 | Placenta Specific 8 |
| CSF1R | 1.2148 | 1.11E-21 | Colony Stimulating Factor 1 Receptor |
| C3 | 1.2111 | 3.38E-08 | Complement Component 3 |
| LAPTM5 | 1.2080 | 1.07E-18 | Lysosomal Protein Transmembrane 5 |
| ITGB2 | 1.2020 | 3.40E-16 | Integrin Subunit Beta 2 |
| CYBB | 1.1947 | 6.71E-17 | Cytochrome B-245 Beta Chain |
| CCL4 | 1.1931 | 4.60E-12 | C-C Motif Chemokine Ligand 4 |
| NETO2 | 1.1845 | 4.76E-24 | Neuropilin And Tolloid Like 2 |
| ACTA2 | 1.1795 | 1.50E-20 | Actin Alpha 2, Smooth Muscle |
| GATA3 | 1.1776 | 2.10E-22 | Gata Binding Protein 3 |
| NCF2 | 1.1640 | 5.92E-12 | Neutrophil Cytosolic Factor 2 |
| LPAR6 | 1.1563 | 1.23E-34 | Lysophosphatidic Acid Receptor 6 |
| C3AR1 | 1.1519 | 5.05E-16 | Complement Component 3A Receptor 1 |
| PYCARD | 1.1434 | 8.15E-21 | Pyd And Card Domain Containing |
| HCLS1 | 1.1424 | 3.33E-25 | Hematopoietic Cell-Specific Lyn Substrate 1 |
| COL15A1 | 1.1259 | 4.20E-11 | Collagen Type Xv Alpha 1 Chain |
| SOX17 | 1.1221 | 2.78E-31 | Sry-Box 17 |
| PHLDA2 | 1.1119 | 2.04E-13 | Pleckstrin Homology Like Domain Family A Member 2 |
| MS4A6A | 1.0619 | 3.71E-13 | Membrane Spanning 4-Domains A6A |
| IDO1 | 1.0566 | 3.15E-09 | Indoleamine 2,3-Dioxygenase 1 |
| GZMA | 1.0495 | 9.17E-15 | Granzyme A |
| CSTA | 1.0485 | 1.66E-11 | Cystatin A |
| TMSB15A | 1.0235 | 2.11E-09 | Thymosin Beta 15A |
| GPR65 | 1.0180 | 2.78E-20 | G Protein-Coupled Receptor 65 |
| TAGLN | 1.0155 | 1.44E-13 | Transgelin |
| COL1A1 | 1.0111 | 1.98E-10 | Collagen Type I Alpha 1 |
| LTF | 1.0108 | 6.03E-09 | Lactotransferrin |
| DNAJB1 | -1.0037 | 2.38E-11 | Dnaj Heat Shock Protein Family (Hsp40) Member B1 |
| PRODH2 | -1.0070 | 3.29E-13 | Proline Dehydrogenase 2 |
| TIPARP | -1.0078 | 1.05E-22 | Tcdd Inducible Poly(Adp-Ribose) Polymerase |
| NFIL3 | -1.0292 | 1.07E-19 | Nuclear Factor, Interleukin 3 Regulated |
| CYP3A7 | -1.0302 | 2.15E-17 | Cytochrome P450 Family 3 Subfamily A Member 7 |
| PBLD | -1.0311 | 2.16E-15 | Phenazine Biosynthesis Like Protein Domain Containing |
| RBP4 | -1.0331 | 3.29E-06 | Retinol Binding Protein 4 |
| UGT2A3 | -1.0347 | 1.26E-13 | Udp Glucuronosyltransferase Family 2 Member A3 |
| SDC1 | -1.0393 | 1.28E-13 | Syndecan 1 |
| ALDH6A1 | -1.0444 | 3.24E-14 | Aldehyde Dehydrogenase 6 Family Member A1 |
| ZFP36 | -1.0582 | 4.00E-19 | Zfp36 Ring Finger Protein |
| SLC22A11 | -1.0620 | 5.17E-14 | Solute Carrier Family 22 Member 11 |
| CD69 | -1.0832 | 1.11E-11 | Cd69 Molecule |
| FMO1 | -1.0833 | 8.22E-09 | Flavin Containing Monooxygenase 1 |
| BHMT | -1.0885 | 3.24E-10 | Betaine--Homocysteine S-Methyltransferase |
| SLC47A1 | -1.0891 | 6.40E-10 | Solute Carrier Family 47 Member 1 |
| SLC2A2 | -1.0945 | 7.13E-12 | Solute Carrier Family 2 Member 2 |
| SLC13A3 | -1.0994 | 3.72E-13 | Solute Carrier Family 13 Member 3 |
| UMOD | -1.1043 | 0.000339144 | Uromodulin |
| DPEP1 | -1.1057 | 1.22E-11 | Dipeptidase 1 (Renal) |
| HRG | -1.1082 | 2.00E-18 | Histidine Rich Glycoprotein |
| EGF | -1.1097 | 3.01E-11 | Epidermal Growth Factor |
| CXCL14 | -1.1100 | 1.63E-08 | C-X-C Motif Chemokine Ligand 14 |
| CEBPD | -1.1191 | 1.89E-16 | Ccaat/Enhancer Binding Protein Delta |
| HSD11B2 | -1.1224 | 9.51E-07 | Hydroxysteroid 11-Beta Dehydrogenase 2 |
| APOM | -1.1297 | 5.97E-12 | Apolipoprotein M |
| ALDH8A1 | -1.1366 | 1.00E-11 | Aldehyde Dehydrogenase 8 Family Member A1 |
| FBP1 | -1.1373 | 4.17E-14 | Fructose-Bisphosphatase 1 |
| NR4A1 | -1.1397 | 6.49E-15 | Nuclear Receptor Subfamily 4 Group A Member 1 |
| DUSP1 | -1.1602 | 6.62E-23 | Dual Specificity Phosphatase 1 |
| CRYM | -1.1621 | 7.01E-16 | Crystallin Mu |
| CYP4F2 | -1.1628 | 3.57E-10 | Cytochrome P450 Family 4 Subfamily F Member 2 |
| LRRC19 | -1.1645 | 7.70E-13 | Leucine Rich Repeat Containing 19 |
| BBOX1 | -1.1680 | 8.81E-10 | Gamma-Butyrobetaine Hydroxylase 1 |
| PIPOX | -1.1684 | 8.60E-13 | Pipecolic Acid And Sarcosine Oxidase |
| BHMT2 | -1.1704 | 1.70E-14 | Betaine--Homocysteine S-Methyltransferase 2 |
| GATM | -1.1818 | 2.15E-14 | Glycine Amidinotransferase |
| SLC19A2 | -1.1851 | 1.93E-32 | Solute Carrier Family 19 Member 2 |
| MGAM | -1.2001 | 4.27E-12 | Maltase-Glucoamylase |
| KCNK5 | -1.2011 | 1.09E-21 | Potassium Two Pore Domain Channel Subfamily K Member 5 |
| SLC22A6 | -1.2055 | 4.58E-12 | Solute Carrier Family 22 Member 6 |
| SLC13A1 | -1.2067 | 3.02E-12 | Solute Carrier Family 13 Member 1 |
| GBA3 | -1.2163 | 2.63E-15 | Glucosylceramidase Beta 3 (Gene/Pseudogene) |
| ALDOB | -1.2192 | 3.85E-14 | Aldolase, Fructose-Bisphosphate B |
| SLC17A1 | -1.2326 | 6.63E-15 | Solute Carrier Family 17 Member 1 |
| ACE2 | -1.2347 | 3.76E-12 | Angiotensin I Converting Enzyme 2 |
| CLDN8 | -1.2353 | 9.08E-08 | Claudin 8 |
| GLYAT | -1.2425 | 3.27E-13 | Glycine-N-Acyltransferase |
| GDF15 | -1.2692 | 1.04E-11 | Growth Differentiation Factor 15 |
| CUBN | -1.2951 | 9.92E-12 | Cubilin |
| APOH | -1.2982 | 7.87E-18 | Apolipoprotein H |
| DEFB1 | -1.3022 | 1.17E-07 | Defensin Beta 1 |
| HAO2 | -1.3099 | 1.04E-11 | Hydroxyacid Oxidase 2 |
| G6PC | -1.3124 | 2.57E-19 | Glucose-6-Phosphatase Catalytic Subunit |
| AFM | -1.3153 | 2.75E-16 | Afamin |
| ATF3 | -1.3457 | 9.99E-14 | Activating Transcription Factor 3 |
| NR4A2 | -1.3464 | 6.37E-21 | Nuclear Receptor Subfamily 4 Group A Member 2 |
| PDK4 | -1.3779 | 2.39E-27 | Pyruvate Dehydrogenase Kinase 4 |
| XPNPEP2 | -1.3816 | 3.41E-14 | X-Prolyl Aminopeptidase 2 |
| PAH | -1.3858 | 1.11E-13 | Phenylalanine Hydroxylase |
| RNF186 | -1.4220 | 1.48E-22 | Ring Finger Protein 186 |
| SLC22A8 | -1.4366 | 7.98E-17 | Solute Carrier Family 22 Member 8 |
| SLC7A9 | -1.4575 | 1.56E-13 | Solute Carrier Family 7 Member 9 |
| SLC17A3 | -1.4775 | 1.95E-13 | Solute Carrier Family 17 Member 3 |
| EGR1 | -1.5039 | 9.21E-15 | Early Growth Response 1 |
| DIO1 | -1.5084 | 2.60E-12 | Deiodinase, Iodothyronine Type I |
| PLG | -1.5151 | 7.03E-12 | Plasminogen |
| FABP1 | -1.5271 | 1.44E-16 | Fatty Acid Binding Protein 1 |
| DPYS | -1.5427 | 1.37E-14 | Dihydropyrimidinase |
| AZGP1 | -1.5479 | 1.80E-13 | Alpha-2-Glycoprotein 1, Zinc-Binding |
| APOLD1 | -1.5579 | 8.27E-13 | Apolipoprotein L Domain Containing 1 |
| SLC27A2 | -1.5722 | 5.51E-16 | Solute Carrier Family 27 Member 2 |
| PCK1 | -1.7270 | 8.79E-19 | Phosphoenolpyruvate Carboxykinase 1 |
| FOS | -1.7671 | 3.14E-09 | Fos Proto-Oncogene, Ap-1 Transcription Factor Subunit |
| GSTA1 | -1.8206 | 1.23E-16 | Glutathione S-Transferase Alpha 1 |
| HPD | -1.8994 | 5.69E-16 | 4-Hydroxyphenylpyruvate Dioxygenase |
| CALB1 | -1.9955 | 6.39E-10 | Calbindin 1 |
| ALB | -2.0997 | 9.07E-18 | Albumin |
| CYP27B1 | -2.1923 | 3.95E-38 | Cytochrome P450 Family 27 Subfamily B Member 1 |
| FOSB | -3.2166 | 1.14E-23 | Fosb Proto-Oncogene, Ap-1 Transcription Factor Subunit |
